# Mediterranean Spontaneously Fermented Sausages: Spotlight on Microbiological and Quality Features to Exploit Their Bacterial Biodiversity

**DOI:** 10.3390/foods10112691

**Published:** 2021-11-03

**Authors:** Federica Barbieri, Giulia Tabanelli, Chiara Montanari, Nicolò Dall’Osso, Vida Šimat, Sonja Smole Možina, Alberto Baños, Fatih Özogul, Daniela Bassi, Cecilia Fontana, Fausto Gardini

**Affiliations:** 1 Department of Agricultural and Food Sciences, University of Bologna, 47521 Cesena, Italy; federica.barbieri16@unibo.it (F.B.); nicolo.dallosso2@unibo.it (N.D.); fausto.gardini@unibo.it (F.G.); 2 Department of Agricultural and Food Sciences, University of Bologna, 40127 Bologna, Italy; 3 Interdepartmental Center for Industrial Agri-Food Research, University of Bologna, 47521 Cesena, Italy; chiara.montanari8@unibo.it; 4 University Department of Marine Studies, University of Split, 21000 Split, Croatia; vida@unist.hr; 5 Department of Food Science and Technology, Biotechnical Faculty, University of Ljubljana, 1000 Ljubljana, Slovenia; sonja.smole-mozina@bf.uni-lj.si; 6 Department of Microbiology, DOMCA S.A.U., 18620 Alhendín, Spain; abarjona@domca.com; 7 Department of Seafood Processing Technology, Faculty of Fisheries, Cukurova University, Adana 01330, Turkey; fozogul@cu.edu.tr; 8 Department for Sustainable Food Process (DISTAS), Università Cattolica del Sacro Cuore, 26100 Cremona, Italy; daniela.bassi@unicatt.it (D.B.); cecilia.fontana@unicatt.it (C.F.)

**Keywords:** natural fermentation, dry fermented sausages, microbial biodiversity, lactic acid bacteria, CNC, 16S metagenomics

## Abstract

The wide array of spontaneously fermented sausages of the Mediterranean area can represent a reservoir of microbial biodiversity and can be an important source of new technological and functional strains able to preserve product properties, counteracting the impoverishment of their organoleptic typical features due to the introduction of commercial starter cultures. We analysed 15 artisanal salamis from Italy, Spain, Croatia and Slovenia to evaluate the microbiota composition, through culture-dependent and culture-independent techniques (i.e., metagenomic analysis), chemical–physical features, biogenic amines and aroma profile. The final pH varied according to origin and procedures (e.g., higher pH in Italian samples due to long ripening and mold growth). Lactic acid bacteria (LAB) and coagulase-negative cocci (CNC) were the dominant population, with highest LAB counts in Croatian and Italian samples. Metagenomic analysis showed high variability in qualitative and quantitative microbial composition: among LAB, *Latilactobacillus sakei* was the dominant species, but *Companilactobacillus* spp. was present in high amounts (45–55% of the total ASVs) in some Spanish sausages. Among staphylococci, *S. epidermidis*, *S. equorum*, *S. saprophyticus*, *S. succinus* and *S. xylosus* were detected. As far as biogenic amines, tyramine was always present, while histamine was found only in two Spanish samples. These results can valorize the bacterial genetic heritage present in Mediterranean products, to find new candidates of autochthonous starter cultures or bioprotective agents.

## 1. Introduction

Fermented meat products represent an important industrial sector in Europe, particularly in the Mediterranean countries (MC). In addition to the economic importance of this supply chain, the cured products constitute a valued cultural heritage strongly linked to the identity of a population or specific production areas. Particularly, a wide variety of sausages are produced using typical regional recipes and ancient processes [1,2]. Many authors reported that the microbiological features and technological attributes of fermented foods, including dry fermented sausages, are affected by the geographical origin, due to the specific manufacturing process conditions and different raw materials and formulations [3,4,5,6,7].

For years, sausages have undergone massive technological, economic, social, and even nutritional transformations. Innovations in the meat industry, meeting production and safety standards, helped to reduce waste and save production time, energy, and costs [1].

Particularly, in the last decades, the use of starter cultures has been introduced in the meat industry to guide fermentation, enhancing product safety but losing biodiversity [8]. However, in the different areas of the MC, the presence of numerous local products still obtained through spontaneous fermentation is recognized as a formidable treasure chest of unexplored microbial biodiversity.

Spontaneous fermentation relies on the presence of indigenous microorganisms conferring peculiar characteristics to the products in terms of both technological and organoleptic traits [9]. The meat environment and the process applied to facilitate the growth of some microbial groups, among which lactic acid bacteria (LAB) and coagulase-negative cocci (CNC) play a major role. Besides, filamentous fungi and yeasts can exert important effects, preventing excessive dehydration and the oxidation of the lipid fraction due to oxygen [9]. The development of the peculiar sausage flavor, aroma and texture relies on the interactions among microorganisms, raw materials and processing technology, due to biochemical and physicochemical reactions in which several bacteria, yeast and fungi cooperate within the meat matrix and its surface [10,11].

The study of traditional spontaneously fermented product characteristics and their microbiota can be a strategy to explore new technological and functional strains and to add value to local productions while preserving authenticity and traditional features. In this context, producers in the rural areas of MCs could take advantage of competitive opportunities and development, safeguarding the traceability and diversification of fermented sausages.

This work aimed to generate new knowledge about the microbial ecology in spontaneously fermented sausages produced in four MCs (Italy, Spain, Croatia, and Slovenia), using a combination of cultivation-dependent and metagenomic techniques. In addition, the safety (i.e., biogenic amine content) and chemical–physical features of the products, as well as their aroma profile, were evaluated.

## 2. Materials and Methods

### 2.1. Sample Collection

A total of fifteen samples of artisanal fermented sausages, produced without starter addition, were collected at the end of production from four different MCs: two samples of Italian salami produced by two different small companies in the Marche region (IM1 and IM2); one salame Alfianello coming from the Lombardia region (IAL); two traditional Slovenian smoked salami (SN and SWO, that differed only for the presence of nitrate/nitrite); seven traditional salchichón and chorizo produced in different locations from Andalusia (Spain): a salchichón Alhendín (ESA), a salchichón Bérchules (ESB), a salchichón Écija (ESE), a salchichón Olvera (ESO), a chorizo Bérchules (ECB), a chorizo Écija (ECE), and a chorizo Olvera (ECO); and three samples from Croatia, a traditional unsmoked salami (HNS), a traditional smoked salami (HS) and a Salami Zminjska Klobasica (HZK).

### 2.2. pH and Composition Analysis

The pH of the 15 ripened samples was detected by using a pH-meter Basic 20 (Crison Instruments, Barcelona, Spain). Aw detection was performed with an Aqualab CX3-TE (Labo- Scientifica, Parma, Italy). All measures were performed in triplicate. A FoodScan instrument (Foss, Hilleroed, Denmark) was used to carry out the sausage centesimal composition. This technique uses near-infrared transmission (NIT), with a wavelength between 850–1050 nm, for inhomogeneous samples. The absorbance data obtained are processed with a mathematical function and a calibration model to calculate the expected value to provide the % of fat, moisture, protein, collagen and salt.

### 2.3. Microbial Counts

For bacterial enumeration, the samples were prepared by removing aseptically the casing, and a slice of approx. 10 g of the sausage was transferred into a stomacher bag, mixed with 90 mL of 0.9% (*w*/*v*) NaCl sterile solution and homogenized in a Lab Blender Stomacher (Seward Medical, London, UK) for 2 min. Appropriate decimal dilutions were prepared and plated onto selective media: (i) Mannitol Salt Agar (MSA) for enumeration of CNC (30 °C for 72 h); (ii) de Man-Rogosa-Sharpe (MRS) agar for LAB (30 °C for 48 h in anaerobic conditions); (iii) Slanetz and Bartley medium for enterococci (44 °C for 24 h); (iv) Sabouraud Dextrose Agar, added with 200 mg/l of chloramphenicol for yeasts and molds (28 °C for 72 h); (v) pour plating on Violet Red Bile Glucose Agar for *Enterobacteriaceae* (37 °C for 24 h); (vi) Violet Red Bile Agar with MUG (4-methylumbelliferyl-ß-D-glucuronide) for the enumeration of *Escherichia coli* (42 °C for 24 h). All media were provided by Oxoid (Basingstoke, UK). The results reported are the means of 3 different sausages. Each sausage was sampled in triplicate.

The presence of *Listeria monocytogenes* and *Salmonella* was evaluated according to the methods EN ISO 11290–1 and EN ISO 6579, respectively [12,13].

### 2.4. DNA Extraction and Sequencing

Total genomic DNA was directly extracted from 200 mg of frozen sausage samples, which were treated with lysozyme at 37 °C for 1 h, followed by mechanic lysis through TissueLyser II (Qiagen) with a frequency of 30 Hz for 5 min. The DNA was then purified using a Wizard genomic DNA purification kit (Promega, Mannheim, Germany) according to the manufacturer’s recommendations. The purified DNA resuspended in water was quantified using a Qubit 4 Fluorimeter (ThermoFisher Scientific, Waltham, MA, USA). After the normalization of DNA concentration, the sequencing was carried out through the Illumina MiSeq platform, generating 300 bp pair-end sequencing reads. The library for Illumina sequencing was generated from V3–V4 variable regions of ribosomal 16S rRNA to characterize the bacterial population of the samples.

### 2.5. Bioinformatic Analysis

FASTQ sequence files from Illumina reads were analysed using DADA2 version 1.8 [14] with the R 3.5.1 environment, which implements a new quality-aware model of Illumina amplicon errors without constructing OTUs [14].

The parameters applied, as described in https://benjjneb.github.io/dada2/tutorial.html (access date on 26 July 2021), were the following: trimLeft equal to 30 and truncLen option set to 270 and 200 for the forward and reverse fastq files, respectively. The comparison between the amplicon sequence variant (ASV) predicted from DADA2 against SILVA database (version 138 updated according to the reclassification of the genus *Bacillus* and *Lactobacillus*) was used for the taxonomic assignment. ASVs belonging to taxa classified as external sample [15] contamination were not included in the composition analysis for microbial population or for ASVs with low abundance setting, specifically a threshold of relative abundance equal to 0.5%. The assignment at the species level for the remaining ASVs was confirmed.

### 2.6. Biogenic Amine Determination

The samples were extracted with trichloroacetic acid, according to Pasini et al. [16]. The extracts were subjected to a dansyl chloride derivatization (Sigma Aldrich, Gallarate, Italy), according to Martuscelli et al. [17]. An HPLC Agilent Technologies 1260 Infinity with the automatic injector (G1329B ALS 1260, loop of 20 µL), equipped with a UV detector (G1314F VWD 1260) set at 254 nm, was used. For the chromatographic separation a C18 Waters Spherisorb ODS-2 (150 × 4.6 mm, 3 µm) column was used with the following gradient elution: 0–1 min acetonitrile/water 35:65, 1–6 min acetonitrile/water 55:45, 6–16 min acetonitrile/water 60:40, 16–24 min acetonitrile/water 90:10, 24–35 min acetonitrile/water 90:10, 35–40 min acetonitrile/water 35:65, 40–45 min acetonitrile/water 35:65, all at a flow rate 0.6 mL/min.

The amounts of amines were expressed as mg/kg with reference to a calibration curve obtained through aqueous dansyl-chloride-derivatized amine standards of concentrations ranging from 10 to 200 mg/L (Sigma-Aldrich, Milano, Italy). The detection limit for all the amines was 3 mg/kg of the sample under the adopted conditions. All the analyses were performed in triplicate.

### 2.7. Aroma Profile Analysis

Gas-chromatography-mass spectrometry coupled with the solid-phase microextraction (GC-MS-SPME) technique was employed for sausage volatile organic compound (VOCs) analysis. A total of 3 g of samples were combined with a known amount of 4-methyl-2-pentanol (Sigma-Aldrich, Steinheim, Germany) as internal standard and analysed according to the protocol reported by Montanari et al. [18]. Volatile peak identification was carried out using Agilent Hewlett–Packard NIST 2011 mass spectral library (Gaithersburg, MD, USA) [19].

The mass spectrum identification was confirmed in the same conditions by the injection of the pure standards (Sigma- Aldrich, St. Louis, MO, USA). Data are expressed as the ratio between each molecule’s peak area and the peak area of internal standard and are the mean of six determinations for each sample.

### 2.8. Statistical Analysis

Principal component analysis (PCA) and cluster analysis (LDA) were carried out using Statistica 8.0 (StatSoft Inc., Tulsa, OK, USA).

## 3. Results and Discussion

### 3.1. Geographical Origin and Manufacturing Processes

The dry-fermented sausages in the study were produced according to traditional recipes with the addition of salt and different spices. The peculiar characteristics of the fifteen products are described in Table 1. The sausage diameter varied between 2.5 and 6 cm. All products were obtained using only pork meat and fat, apart from two Slovenian samples (SN and SWO) that also contained 20% of beef meat. The ripening conditions (temperature, time etc.) differed between the samples, and only three samples were smoked, i.e., two Slovenian samples (SN and SWO) and a Croatian sample (HS sample).

### 3.2. Physico-Chemical Characterization

In Table 2 the chemical–physical parameters of the fermented sausages at the end of ripening are shown. Wide differences in the final pH were observed, with values ranging from a minimum of 4.52 in the ECO samples to a maximum of 6.42 in IM1.

In general, Italian products have the highest pH values (between 5.88 and 6.42), together with Croatian ones (between 5.72 and 6.05). On the other hand, Slovenian sausages showed lower pH values (5.20 and 5.39). These values agree with those reported by Lešić et al. [20] for Croatian and Slovenian sausages and with the data reported by Cardinali et al. [21] for Italian Fabriano sausages.

Slovenian products are probably more subjected to northern European production influences. In fact, northern products are generally dried sausages with a pH around or even below 5 and often undergo a smoking phase (that inhibits molds), while Mediterranean sausages are usually long-ripened and with pH values up to 6.2–6.4, given the possible growth of desirable molds [22,23].

The Spanish fermented products were widely different in terms of pH, due to the heterogeneous traditional manufacturing process developed in the whole country. In fact, chorizo samples showed a very low pH (between 4.52 and 5.04), while the salchichón had a pH ranging from 5.13 to 5.83. There are different set of data reported in the literature for chorizo characteristics, and among them the pH can vary: results of Galician chorizo showed a pH value of about 5.6 [24], while Asturian products had pH between 5.0 and 5.1 [25].

Great variability was also observed in the final a_w_ of the products. In some case, this parameter reached extremely low values, such as in IM2 (0.760), ESB (0.811), IM1 (0.824) and SN (0.823). On the other hand, HNS showed the highest value (0.928). In any case, the aw values reflected the water content of the final products. This parameter ranged from 20.42% in IM2 to 39.04% in ESO. The salt content in the final products ranged from 2.94 (HNS) to 4.48 (IM2).

### 3.3. Microbiological Analysis

Microbial counts were performed to enumerate in the final product LAB, CNC, enterococci, enterobacteria (including *E. coli*) and yeasts (Table 3).

The largest microbial population was generally represented by LAB, whose counts ranged from 4.4 (ECB) to 8.7 (HNS) log cfu/g. The highest LAB counts were found in samples collected from Croatia and Italy. The lowest numbers were associated with sausages from Spain, particularly ECB and ECE. LAB have been described as the main bacterial population in the dominant microbiota of Croatian traditionally fermented sausages and in Italian spontaneously fermented salami, with loads of 7–8 log cfu/g in the final products [26,27,28].

The counts of CNC were higher in Italian Fabriano salami (7.1 log cfu/g) and in HZK (7.2 log cfu/g). This microbial group was below the detection limit in SN, ECB, ECE and ECO, while in SWO, its concentration was very low (1.4 log cfu/g). The presence of CNC was strongly related to pH, and in particular, they seem to be inhibited by lower pH.

Enterococci were not detected or were detected in sporadic quantities (<1 log cfu/g) in Italian and Slovenian samples and in three Spanish samples (ESA, ECB, ECO). The highest counts of this microbial group were found in Croatian samples (3.2–4.8 log cfu/g).

Yeasts ranged from 2.3 to 5.4 log cfu/g, except for SN sausage. This microbial population can have an important role in sausage-ripening, contributing to the formation of the aroma and to the evolution of product sensory features [29,30].

*Enterobacteriaceae* were present in detectable amounts only in Croatian products, with values ranging between 2.7 (HS) and 5.1 (HZK). In all other samples, this population was below the limit of determination. High levels of *Enterobacteriaceae* can indicate the low microbiological quality of the product, and high concentrations (>4 log cfu/g) of this population have been enumerated in several ripened traditional products [31,32,33].

The counts of *E. coli* were always below the detection limit. In addition, a search for pathogenic microorganisms such as *Listeria monocytogenes* and *Salmonella* was carried out, and no positive samples were found.

### 3.4. Metagenomic Analysis

A more detailed picture of the bacterial composition of spontaneously fermented sausage was obtained through amplicon sequencing and metagenomic analysis.

Only species and genera that reached a concentration higher than 0.5% of amplicon sequence variants (ASVs) in at least one of the samples were considered. A total of more than 500 ASVs were detected, indicating very high biodiversity in the composition of the microbiota of the European salamis taken into consideration. The relative abundance of the ASVs attributed to genera or species is given in Table 4.

Beyond a large number of microbial species, their relative composition was also very variable among the products, even for those collected in the same geographical area.

While in some samples most of the ASVs were attributed to a single group/species, some sausages were characterized by higher biodiversity, with an important diversification in the microbiota composition (e.g., HZK and ESO).

Among LAB, several genus and species were present. Members of the genus *Latilactobacillus* were found in all the sausages. *Latilactobacillus sakei* was the dominant species (>50% of ASVs in IM2, IAL, and SN). The *Latilactobacillus sakei* group (which included *Lat. curvatus*, *Lat. graminis* and *Lat. fuchuensis*) was found in all the sausages, even if in lower percentage, except for IM1 and ESA. *Lat. sakei*, and to a lesser extent *Lat. curvatus*, have been reported as the prevailing LAB species in fermented meat products originating from France, Italy and Spain [6,34,35]. In fact, LAB species diversity of fermented sausages is known to be limited, being *Lat. sakei*-predominant during the ripening process, due to the species’ excellent adaptation, competitiveness and assertiveness in the meat matrix [36,37,38,39,40,41]. This predominance over other LAB can be attributed to its salt-tolerant and psychrotrophic nature and its specialization in metabolic pathways in the meat environment, including the arginine deiminase pathway and the utilization of nucleosides [36,42,43,44,45,46].

Other lactobacilli were sporadically detected in low amounts, such as *Lacticaseibacillus casei* group in ESB, *Lactiplantibacillus plantarum* group in ESO, *Ligilactobacillus* sp. and *Loigolactobacillus rennini* in HS and *Dellaglioa algida* in SWO. High levels of the member of the genus *Companilactobacillus* (*Com. alimentarius*, *Com. heilongjiangensis* and *Com. versmoldensis*) were present in many Spanish sausages, in particular ESE and ECB, in which they represented 55.3 and 45.0% of the total ASVs. The abundance of *Companilactobacillus* found only in Spanish sausages, is a regional peculiarity already reported in the literature [47,48]. Moreover, these species have been isolated as spoilage organisms in ready-to-eat meats and from other dry fermented sausages originating from Belgium and Italy [6,49,50] and have been proposed as autochthonous probiotic starter cultures for meat products [51].

Heterofermentative lactobacilli were present only in ESO and ECE (*Levilactobacillus yonginensis* group and *Limosilactobacillus mucosae*, respectively). In addition, ASVs belonging to some dairy LAB (*Lactobacillus helveticus*, *Streptococcus thermophilus* and *Lactococcus* sp.) were found in some Spanish products. These species can be related to the use of powdered milk or other dairy derivatives during manufacturing. The heterofermentative cocci were represented by *Leuconostoc carnosum*, present in relevant percentages in IM1 (15,8%), SN (19.1%), SWO (23.3%) and HZK (14.8%); *Leuconostoc* sp. (17.6% in IAL and 15.6% in SWO); and *Weissella* sp., found only in ESO.

Five species of staphylococci were detected (*Staph. epidermidis*, *Staph. equorum*, *Staph. saprophyticus*, *Staph. succinus* and *Staph. xylosus*). They were found only in some sausages, and they were dominant in IM1 (67.7% of ASVs belonging to *Staph. equorum* and 1.5% to *Staph. succinus*) and ESA (98.1 of ASVs belonging to *Staph. xylosus*).

Among CNS, *Staphylococcus xylosus* is the prevalent species associated with salamis, even if greater species diversity can be found, *Staphylococcus epidermidis*, *Staphylococcus equorum* and *Staphylococcus saprophyticus* also being reported in these products [52,53,54].

Staphylococci exert several important technological roles in sausage production, such as contribution to flavor and color [54,55], and since they are able to use alternative energy sources, such as arginine and nucleoside, their ecological persistence is assured [37]. These bacteria were detected as subdominant fractions in several fermented meat products, and their presence was associated with mammal skin, being competitive in the acidic and anaerobic conditions prevailing during meat fermentation [56,57]. It is noteworthy that many samples showed a very low pH (i.e., Spanish products) that could have hindered their growth. The results therefore underline the sensitivity of this genus to low pH, even in a potentially favorable environment, thanks to the low aw values. These populations probably suffered the strong initial acidification in these products, which was not followed by a significant increase in pH (scarcity of molds, etc.).

*Carnobacterium* sp. was highlighted only in some Croatian samples, i.e., HS and HNS, albeit with a relevant percentage (19.11% and 9%, respectively). This genus has been found in Fabriano-like products [21], and it has been associated with meat spoilage phenomena [58].

Among *Actinobacteria*, *Kocuria* sp. and *Corynebacterium* sp. were present only in HZK, while *Brevibacterium casei* and *Rothia* sp. were present in ECE.

The meat spoiler *Brochothrix thermosphacta* was detected in several sausages, and its concentration was particularly relevant in HZK (19.58%), IM2 (14.91%) and ESE (14.23%). This species has been reported to be part of the Fabriano-like sausage microbiota by Cardinali et al. [21], and it is commonly associated with spoiled meat-based products [59,60].

Among Gram-negative bacteria, *Pseudomonas* sp. appeared at a high concentration in the Slovenian samples, in the Spanish ESE (16.7% of ASV) and especially in HNS, in which this genus represented 26.8% of ASVs. Enterobacteria (*Klebsiella* sp. and *Escherichia*/*Shigella* group) were found in ESB, ESO and ECE.

**Figure 1:**
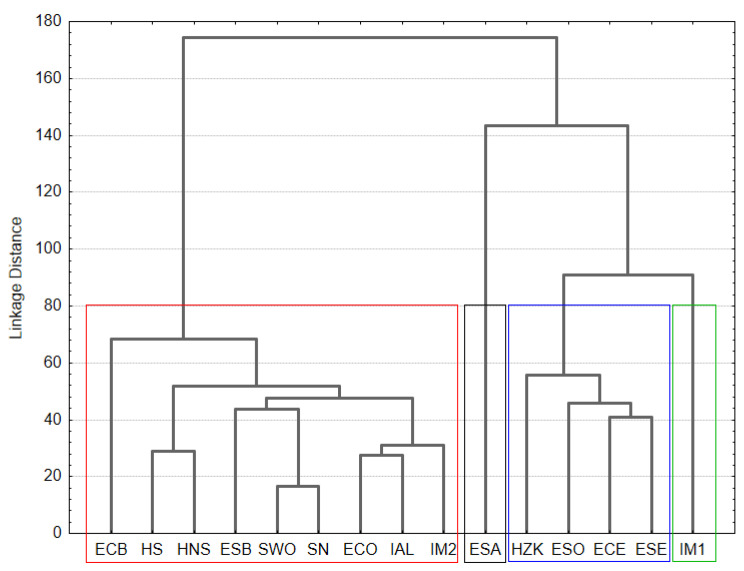
Tree diagram for the similarity of sausage metagenomic analysis obtained using Euclidean distances as distance metric and Ward’s method for amalgamation rule.

To better evidence the differences in the ASV composition, a cluster analysis was carried out. The results are reported in Figure 1.

Four different clusters were obtained. The first contained only the Italian sausage IM1. In the second cluster were grouped three Spanish sausages (ESE, ECE and ESO) and the Croatian sample HZK. The third cluster was represented only by ESA. The fourth cluster included nine samples, and in particular the Spanish sausages ECB, ECO and ESB; the Croatian HNS and HS; the Slovenian sausages; and the Italian samples IM2 and IAL.

**Figure 2:**
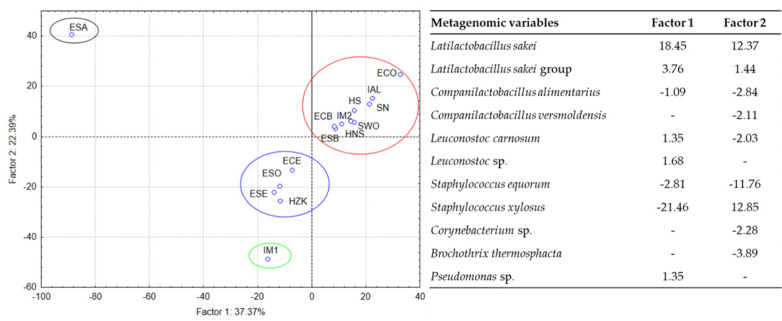
PCA factor coordinates for the first two factors explaining the variability of the microbial populations of the sausages according to the metagenomic analysis. The factor coordinates of the most relevant metagenomic variables (characterized by values higher than |1|) for the first two factors are also reported, which represent the projection of the metagenomic variables on the factor-plane.

To highlight the microorganisms responsible for the clustering of sausages, a PCA was applied whose first two components explained 59.76% of the variability (Figure 2).

The grouping of the sausages reflected the results of the tree diagram. In Figure 2, factor coordinates of the metagenomic variables are shown. Only the metagenomic variables with coordinates characterized by an absolute value higher than 1 are reported. The *Lat. sakei* and the *Lat. sakei* group were the main ones responsible for the description of the largest group located in the first quadrant, while *Staph. xylosus* determined the collocation of ESA in the left part of the second quadrant. The position of IM1 depends on the variable *Staph. equorum*, while the remaining group collocation is mainly influenced by the presence of *Companilactobacillus* (especially for the Spanish samples), *Corynebacterium* for the Croatian sausage and *Brochothrix thermosphacta* for all the samples grouped in this cluster.

### 3.5. Biogenic Amine Concentrations

The biogenic amine concentrations in the sausages are reported in Table 5.

Tyramine was present in all the samples, with concentrations ranging from 47.7 mg/kg (IM2) to 366.8 mg/kg (HNS). The mean content of this biogenic amine was 165.5 mg/kg, with a standard deviation of 88.8 mg/kg, indicating a fair variability among the fifteen dry fermented sausages. In general, the Italian products showed lower tyramine concentrations, while the highest amounts were found in the Croatian salamis HNS and HS. These amounts are similar to those reported by EFSA [61], which indicated tyramine mean concentration of 136 mg/kg, with the 95th percentile of 397 mg/kg, in 400 European salami samples.

Histamine was found only in two Spanish samples (ESB and ECE) at concentrations of 195.8 and 174.7 mg/kg, respectively. This amine is considered the most dangerous for human health as it can cause various adverse effects known as “histamine poisoning” [62]. The quantities found in the two Spanish samples, although not very high, are nevertheless significant, especially when compared with the maximum amounts allowed in some fish products. These latter are the only ones regulated for histamine presence and generally admit a maximum quantity of 100 mg/kg in fresh fish and of 200 mg/kg for processed products [63].

The presence of putrescine was more variable: in four samples, this amine was not detected, while two Croatian samples (HNS and HS) showed the highest amounts (about 300 mg/kg). In the same samples, higher quantities of cadaverine were also observed.

Both Gram-negative and Gram-positive bacteria have been described as biogenic amine producers, with wide variability in aminobiogenetic potential between different strains of the same species. Spoilage microorganisms such as enterobacteria and pseudomonads can be strong histamine, cadaverine and putrescine accumulators, and biogenic amines produced by these microbial populations can also be found in fermented sausages [64,65]. On the other hand, decarboxylase activity has been found in Gram-positive strains, also belonging to the genus *Staphylococcus* and LAB. While *Lat. sakei* is usually known for its inability to produce BAs, in many other LAB species, strains with decarboxylase activity are present. *Lat. curvatus*, for example, can accumulate both tyramine and histamine as well as *Com. alimentarius* [66,67,68]. The ability to produce BAs has been found in other genera found in this investigation, such as *Leuconostoc* sp., *Weissella* sp. and *Carnobacterium* [68].

### 3.6. Sausage Aroma Profile

The aroma profile of the fifteen ripened sausages has been studied. The unidentified compounds accounted for less than 1% of the total peak area in each sample.

The analysis of the volatile organic compounds (VOCs) allowed the clear differentiation of the samples, reflecting the different formulations and production and ripening conditions traditionally adopted in the Mediterranean geographical areas. Indeed, it has been reported that these differences can influence sausage aroma, being dry fermented sausage flavor affected by many processing factors, i.e., raw materials, spices, microbiota composition, smoking, etc. [55].

Within this wide variability, some common characteristics can be found by grouping the identified molecules in homogeneous chemical groups: ketones, aldehydes, alcohols, acids, and esters (Table 6), as well as molecules deriving from spices and smoking, reported as Supplementary Materials (Tables S1 and S2, respectively).

Concerning the molecules associated with the spices included in the meat batter formulation (Table S1), they belong to terpenes and terpenoids, phenylpropenes and compounds deriving from garlic (dimethyl disulfide, diallyl sulfide, etc.) [69]. These latter compounds are particularly present in Slovenian salami, Spanish chorizo and Croatian products, except for the HNS sample. Among terpenes, D-limonene certainly is the prevalent molecule in products in which pepper has been used. Many other terpenes deriving from this spice, such as myrcene, linalool, copaene, carene, o-cymene, etc. [70], have been detected in these products, while they are absent in chorizo sausages, where oregano has been used. On the other hand, eugenol, safrole and methyl-eugenol are associated with the use of spices such as nutmeg, cinnamon and cloves [71,72].

The VOCs derived from smoke (Table S2) include furans and phenols, already reported for smoked products [73], and their presence is higher in the Slovenian sausages (SN and SWO) and HS, characterized by a smoking phase during their production. Furthermore, some of these VOCs were detected also in the Spanish chorizo samples, due to the traditional use of smoked paprika [74].

**Figure 3:**
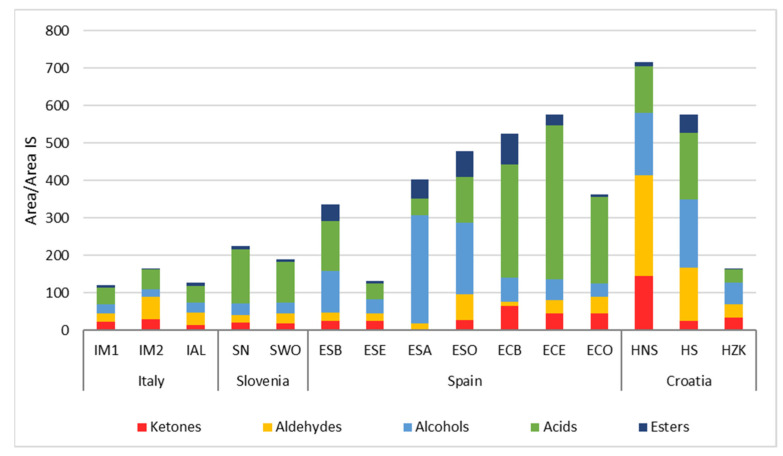
Presence of the different classes of volatile organic compounds (VOCs) in the fifteen fermented sausage samples considered. The values are expressed as the ratio between the peak area of the compound considered and the area of the internal standard.

Table 6 reports VOCs derived from the microbial biochemical activities that occurred during sausage fermentation and ripening. The compounds are grouped into chemical families, whose total amounts are shown in Figure 3. Higher quantities of VOCs were found in some Spanish products and two Croatian samples.

Ketones were evenly distributed in the analysed samples, except for chorizo and HNS samples where their total amount was higher. On the other hand, lower values were found in salchichón ESA. These molecules mostly derive from fatty acid oxidation and, in particular, from β-oxidation. Some microbial groups such as staphylococci and fungi can have a role in these phenomena. It is interesting to observe that products characterized by high concentrations of fat (for example HNS) showed the higher presence of ketones. HS, having the same formulation of HNS but subjected to smoking, presented lower ketone amounts: this can be attributed to the antioxidant role of some of the compounds produced by smoking. It is also interesting to note that chorizo samples are characterized by higher ketone amounts among Spanish products. These fermented sausages did not contain nitrates or nitrites, which exert a well-known antioxidant activity, while these preservatives were present in salchichón formulations.

Among ketones, 2-butanone prevailed in some samples, i.e., ECB and HNS (Table 6). The presence of this molecule in fermented meat products is common but its contribution to aroma profile can be negative depending on its amounts and the balance with other VOCs [75]. Diacetyl (2,3-butanedione) and acetoin (3-hydroxy-2-butanone) are particularly present in some Spanish products (ECB and ECO), in Slovenian samples and in two Croatian sausages (HNS and HZK). Diacetyl and acetoin are mainly produced through the catabolism of pyruvic acid by LAB [10,76].

Aldehydes are particularly relevant in the Italian sausage IM2 and a Spanish salchichón (ESO), but the maximum amounts were found in Croatian HNS and HS. Most of the detected aldehydes can derive from fatty acids oxidation. Within certain limits, these compounds can contribute to the typical sausage aroma profile. Nevertheless, given their strong aroma perception characterized by herbaceous notes, excessive quantities can lead to organoleptic defects (i.e., rancidity) [10]. Hexanal, together with nonanal, is certainly the most characteristic molecule of this VOC group. On the other hand, the methyl-branched aldehydes, such as isovaleric aldehyde (3-methyl-butanal), have often been associated with fermented sausage aroma and can derive from the bacterial metabolism of branched amino acids, in particular leucine [44,77]. Benzaldehyde and benzeneacetaldehyde are present in most of the samples. In particular, HS contained higher amounts of benzeneacetaldehyde, while benzaldehyde is predominant in HNS and ECO. These VOCs are the result of aromatic amino acid (phenylalanine and tyrosine) metabolism and can contribute to the sausage flavor imparting floral and almond notes [78].

Alcohols are present in all the samples, but their amounts were higher in HNS and HS and some Spanish samples, particularly in ESA and ESO samples (Table 6). Among alcohols, ethanol was the most abundant in the samples, with high amounts in some Spanish salchichón (ESB, ESA, ESO) and in Croatian sausages, while a lower presence was highlighted in Italian and Slovenian salamis. The presence of this compound can be influenced by the wine addition in meat batter formulation or it can be the result of several microbial pathways, i.e., pyruvate or amino acid metabolisms [77,79], being therefore strongly influenced by the natural microbiota composition of each product. Phenethyl alcohol, which is the results of benzenacetaldehyde reduction and that can give a rose odor, was present in significant amounts in HS and ESA, samples characterized by high amounts of its precursor.

Acids prevailed in chorizo samples but they were the most represented molecular class in Slovenian sausages (Figure 3). Acetic acid was found in low amounts in Italian salamis and in ESA, ESE and HZK, which showed high pH values. In contrast, significantly higher amounts of acetic acid were detected in the chorizo samples, particularly in ECE. These samples had the lowest pH, ranging from 4.52 to 5.04. Acetic acid can be produced, similarly to ethanol, with many bacterial metabolic pathways and different microbial groups able to be responsible for its accumulation, including LAB, staphylococci and fungi.

It is interesting to underline the presence of isovaleric acid (3-methyl, butanoic acid) (Table 6), whose occurrence in fermented sausages is well-documented and which can exert a very strong organoleptic impact even in low quantities [80,81]. Higher amounts of this VOC have been detected in IM1, while it was not present in very small quantities in HS and HNS.

Esters were present in lower amounts in comparison to other VOC classes and were mainly represented by ethyl acetate, ethyl hexanoate and ethyl octanoate (Table 6). Their levels showed dependence on sausage type. They were more abundant in some Spanish samples and in HS (Figure 3), being related to the presence of their precursors and to the esterase activities typical of the microbial communities forming the microbiome.

## 4. Conclusions

The analysis of the microbial communities associated with traditional spontaneous fermented meat products highlighted the high variability in the qualitative and quantitative composition of the microbiota involved in these natural fermentations. LAB and CNS were the most representative microorganisms in all the sausages. However, their relative ratio drastically changed. In addition, within each group, the relative presence of species and genus was extremely different. From this point of view, LAB was characterized by high biodiversity, and *Latilactobacillus* was the only genus found in all the products. The great LAB biodiversity can derive from both meat and the production environment, affecting the growth and survival of the different microbial groups.

These differences are reflected in the first instance in sausage safety characteristics, i.e., biogenic amine concentrations. Moreover, also the volatilome, and consequently the peculiar sensory traits of traditional products, is dependent on the complexity of the microbiota. In many sausages considered here, increased microbial biodiversity caused VOCs to be more complex, both qualitatively and quantitatively.

In other words, the biodiversity highlighted the need for these microbes to be exploited to find new strain candidates to be used as autochthonous starter cultures or bioprotective agents in meat products. Indeed, this work provided a basin of indigenous LAB strains, belonging to different species, that will be further studied for their features, considering both safety and technological aspects, in order to select the most promising for food applications. Being highly adapted to specific ecological niches, they can be successfully used in traditional meat products to control undesirable microbiota (also avoiding the accumulation of biogenic amines) and/or to endow the final product with peculiar aromatic characteristics. Moreover, they can become an important part of innovation in small-scale productions, enhancing the sustainability and competitiveness of these small industries.

Finally, a deep knowledge of the peculiar characteristics of traditional fermented sausages can valorize these niche productions, guaranteeing their recognizability.

## Figures and Tables

**Table 1:** Main characteristics of sausages collected in the four MCs (Italy, Slovenia, Spain and Croatia) in terms of ingredients, type of casing, presence of preservatives and smoking phase.

Characteristics of the Tested Sausages	Italy			Slovenia		Spain							Croatia		
	IM1	IM2	IAL	SN	SWO	ESA	ESB	ESE	ESO	ECB	ECE	ECO	HNS	HS	HZK
Section	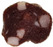	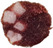	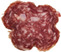	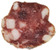	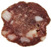	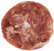	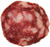	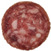	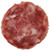	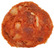	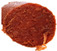	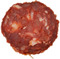	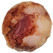	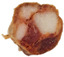	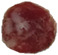
Diameter (cm)	5	3.5	6	5	5	4	5.5	4.5	5.5	4.5	4	5	3.5	3.5	2.5
Type of lean meat	pork	pork	pork	pork/bovine (3:1)	pork/bovine (3:1)	pork	pork	pork	pork	pork	pork	pork	pork	pork	pork
Fat in the meat batter (%)	8–12	8–12	20–30	19	19	20–25	20–30	20–30	20–25	25–30	25–30	15–20	30–35	30–35	20
Fat characteristics	cubes	cubes	minced	cubes	cubes	minced	minced	minced	minced	minced	minced	minced	cubes	cubes	cubes
Spices	pepper, white wine	pepper, white wine	pepper, cinnamon, nutmeg, cloves	pepper, garlic	pepper, garlic	pepper, nutmeg	pepper, nutmeg	pepper, nutmeg	pepper, nutmeg	paprika, oregano, nutmeg, coriander	paprika, oregano, nutmeg, coriander	paprika, oregano, nutmeg, coriander	pepper, garlic, mild paprika, hot paprika	pepper, garlic, mild paprika, hot paprika	pepper, garlic, wine
Nitrate/Nitrite	no	no	yes	yes	no	yes	yes	yes	yes	no	no	no	no	no	no
Type of casing	natural	natural	natural (pork or bovine)	collagen	collagen	collagen	collagen	collagen	collagen	collagen	collagen	collagen	natural (pork intestine)	natural (pork intestine)	natural (pork intestine)
Smoking	not smoked	not smoked	not smoked	smoked	smoked	not smoked	not smoked	not smoked	not smoked	not smoked	not smoked	not smoked	not smoked	smoked	not smoked

**Table 2:** Chemical-physical parameters of the fifteen dry fermented sausages at the end of ripening.

	Italy			Slovenia		Spain							Croatia		
	IM1	IM2	IAL	SN	SWO	ESA	ESB	ESE	ESO	ECB	ECE	ECO	HNS	HS	HZK
pH	6.42 ± 0.02	6.09 ± 0.01	5.88 ± 0.03	5.20 ± 0.02	5.39 ± 0.01	5.83 ± 0.03	5.63 ± 0.04	5.80 ± 0.02	5.13 ± 0.03	4.77 ± 0.02	5.04 ± 0.01	4.52 ± 0.03	5.81 ± 0.05	5.72 ± 0.04	6.05 ± 0.03
a_w_	0.824 ± 0.003	0.760 ± 0.002	0.879 ± 0.002	0.823 ± 0.002	0.832 ± 0.003	0.917 ± 0.002	0.811 ± 0.002	0.848 ± 0.003	0.911 ± 0.004	0.895 ± 0.001	0.870 ± 0.003	0.908 ± 0.001	0.928 ± 0.001	0.903 ± 0.003	0.890 ± 0.001
Humidity (%)	26.15 ± 0.35	20.42 ± 0.38	30.11 ± 0.40	25.05 ± 0.27	26.07 ± 0.38	38.54 ± 0.19	25.45 ± 0.22	27.60 ± 0.18	39.04 ± 0.44	31.12 ± 0.30	30.25 ± 0.41	38.54 ± 0.29	28.75 ± 0.33	25.11 ± 0.38	32.69 ± 0.23
Fat (%)	34.13 ± 0.31	35.79 ± 0.33	34.18 ± 0.26	40.65 ± 0.17	41.33 ± 0.41	29.75 ± 0.28	42.15 ± 0.30	36.05 ± 0.35	29.42 ± 0.21	43.75 ± 0.25	40.38 ± 0.29	29.71 ± 0.37	50.37 ± 0.40	47.21 ± 0.46	30.21 ± 0.26
Proteins (%)	34.32 ± 0.21	37.73 ± 0.35	29.27 ± 0.17	27.61 ± 0.60	26.15 ± 0.44	26.47 ± 0.29	26.83 ± 0.33	28.48 ± 0.24	25.20 ± 0.48	18.44 ± 0.41	22.53 ± 0.26	23.71 ± 0.35	15.14 ± 0.40	20.08 ± 0.29	30.29 ± 0.34
Collagen (%)	1.12 ± 0.05	1.62 ± 0.06	2.61 ± 0.07	3.25 ± 0.03	3.23 ± 0.09	1.02 ± 0.10	1.28 ± 0.04	3.72 ± 0.06	2.10 ± 0.09	3.34 ± 0.08	2.69 ± 0.05	3.61 ± 0.04	2.81 ± 0.11	3.84 ± 0.07	2.08 ± 0.10
Salt (%)	4.31 ± 0.10	4.48 ± 0.05	3.84 ± 0.09	3.44 ± 0.07	3.24 ± 0.11	4.26 ± 0.02	4.24 ± 0.08	4.10 ± 0.06	4.28 ± 0.12	3.31 ± 0.05	4.12 ± 0.04	4.42 ± 0.08	2.94 ± 0.09	3.78 ± 0.05	4.69 ± 0.07

**Table 3:** Concentrations (log cfu/g) of the main microbial groups in the fifteen sausages at the end of ripening.

	Italy			Slovenia		Spain							Croatia		
	IM1	IM2	IAL	SN	SWO	ESA	ESB	ESE	ESO	ECB	ECE	ECO	HNS	HS	HZK
LAB	7.07 ± 0.48	8.52 ± 0.11	8.26 ± 0.16	6.57 ± 0.02	7.28 ± 0.28	7.85 ± 0.75	6.96 ± 0.21	6.32 ± 0.30	7.78 ± 0.14	4.41 ± 0.72	5.88 ± 1.02	7.73 ± 0.27	8.67 ± 0.11	8.43 ± 0.09	8.54 ± 0.03
CNS	7.12 ± 0.09	7.13 ± 0.12	5.22 ± 1.05	<1	1.44 ± 2.03	3.65 ± 0.22	3.05 ± 4.31	4.54 ± 0.16	5.40 ± 0.12	<1	<1	<1	5.34 ± 0.82	5.09 ± 0.01	7.24 ± 0.32
Enterococci	<1	0.60 ± 0.75	0.89 ± 0.71	<1	<1	<1	2.37 ± 0.10	2.19 ± 0.53	2.05 ± 0.38	<1	1.19 ± 0.35	0.89 ± 0.46	3.74 ± 0.12	3.18 ± 0.74	4.80 ± 0.16
Yeasts and molds	5.44 ± 0.05	5.01 ± 0.26	3.46 ± 0.49	0.95 ± 1.35	3.26 ± 0.37	5.34 ± 0.25	3.41 ± 0.80	4.10 ± 0.58	4.59 ± 0.59	3.10 ± 0.27	2.60 ± 0.25	3.70 ± 0.08	2.27 ± 0.31	3.85 ± 0.31	4.15 ± 0.10
*Enterobacteriaceae*	<1	<1	<1	<1	<1	<1	<1	<1	<1	<1	<1	<1	3.55 ± 0.50	2.71 ± 0.82	5.12 ± 0.49

**Table 4:** Relative abundance of amplicon sequence variants (ASVs) in the fifteen sausage samples analysed by metagenomic analysis. Only species and genera which reached a concentration higher than 0.5% in at least one of the samples are reported.

	Italy			Slovenia		Spain							Croatia			
	IM1	IM2	IAL	SN	SWO	ESA	ESB	ESE	ESO	ECB	ECE	ECO	HNS	HS	HZK	
*Latilactobacillus sakei*	9.51	52.71	54.54	55.24	44.75	0.71	36.32	2.89	3.88	41.24	15.12	72.43	35.68	45.36	9.72	
*Latilactobacillus sakei* group	- *	9.55	17.99	4.94	2.34	-	4.85	10.87	23.16	4.95	6.67	22.25	24.63	16.44	2.57	
*Lactiplantibacillus plantarum* group	-	-	-	-	-	-	-	-	2.64	-	-	-	-	-	-	
*Lacticaseibacillus casei* group	-	-	-	-	-	-	1.16	-	-	-	-	-	-	-	-	
*Companilactobacillus alimentarius*	-	-	-	-	-	-	-	21.75	5.81	-	32.32	5.32	-	-	-	
*Companilactobacillus heilongjiangensis*	-	-	-	-	-	-	-	-	1.32	-	-	-	-	-	-	
*Companilactobacillus versmoldensis*	-	-	-	-	-	-	-	33.58	3.96	44.98	2.20	-	-	-	-	
*Ligilactobacillus* sp.	-	-	-	-	-	-	-	-	-	-	-	-	-	4.80	-	
*Loigolactobacillus rennini*	-	-	-	-	-	-	-	-	-	-	-	-	-	7.94	-	
*Dellaglioa algida*	-	-	-	-	3.66	-	-	-	-	3.32	-	-	-	-	-	
*Levilactobacillus yonginensis* group	-	-	-	-	-	-	-	-	3.36	-	-	-	-	-	-	
*Limosilactobacillus mucosae*	-	-	-	-	-	-	-	-	-	-	4.44	-	-	-	-	
*Lactobacillus helveticu*s	-	3.07	-	-	-	-	25.54	-	19.45	0.94	-	-	-	-	-	
*Lactococcus* sp.	-	-	1.12	-	-	-	-	-	-	-	2.72	-	-	-	-	
*Streptococcus* sp.	-	-	-	-	-	-	3.68	-	-	2.09	4.61	-	-	-	-	
*Leuconostoc carnosum*	15.84	-	8.75	19.06	23.26	-	6.70	-	1.70	0.09	-	-	-	-	14.80	
*Leuconosto*c sp.	0.71	-	17.60	2.74	10.20	-	15.63	-	-	-	-	-	-	-	3.65	
*Weissella* sp.	-	-	-	-	-	-	-	-	11.40	-	-	-	-	-	-	
*Carnobacteriu*m sp.	-	-	-	-	-	-	-	-	-	-	-	-	9.00	19.11	-	
*Staphylococcus epidermidis*	-	-	-	-	-	-	-	-	-	-	4.58	-	-	-	-	
*Staphylococcus equorum*	67.74	11.65	-	-	-	-	-	-	4.47	-	-	-	-	-	14.04	
*Staphylococcus saprophyticus*	-	4.20	-	-	-	-	-	-	-	-	-	-	-	-	-	
*Staphylococcus succinus*	1.47	2.09	-	-	-	-	-	-	4.35	-	-	-	-	-	5.27	
*Staphylococcus xylosus*	-	1.81	-	-	-	98.13	-	-	-	2.38	-	-	-	-	-	
*Kocuria* sp.	-	-	-	-	-	-	-	-	-	-	-	-	-	-	1.25	
*Corynebacterium* sp.	-	-	-	-	-	-	-	-	-	-	-	-	-	-	27.91	
*Corynebacterium variabile*	-	-	-	-	-	-	-	-	-	-	-	-	-	-	1.21	
*Brevibacterium casei*	-	-	-	-	-	-	-	-	-	-	1.59	-	-	-	-	
*Rothia* sp.	-	-	-	-	-	-	-	-	-	-	4.10	-	-	-	-	
*Brochothrix thermosphacta*	4.73	14.91	-	1.73	5.81	-	-	14.23	12.77	-	7.90	-	3.91	1.65	19.58	
*Escherichia/Shigella* sp.	-	-	-	-	-	-	1.45	-	-	-	0.68	-	-	-	-	
*Klebsiella* sp.	-	-	-	-	-	-	1.21	-	1.20	-	4.56	-	-	-	-	
*Pseudomona*s sp.	-	-	-	14.64	7.57	-	-	16.68	-	-	1.42	-	26.79	4.70	-	
*Photobacterium piscicola*	-	-	-	-	-	-	1.67	-	-	-	-	-	-	-	-	
*Acinetobacter* sp.	-	-	-	1.64	2.42	1.16	-	-	-	-	2.26	-	-	-	-	
*Erysipelothrix* sp.	-	-	-	-	-	-	1.80	-	0.52	-	4.84	-	-	-	-	

*: not detected.

**Table 5:** Concentrations (mg/kg) of the main biogenic amines detected in the fifteen samples at the end of ripening.

	Italy			Slovenia		Spain							Croatia		
	IM1	IM2	IAL	SN	SWO	ESA	ESB	ESE	ESO	ECB	ECE	ECO	HNS	HS	HZK
Histamine	- *	-	-	-	-	-	195.79 ± 27.29	-	-	-	170.74 ± 28.54	-	-	-	-
Tyramine	73.87 ± 21.83	47.65 ± 12.93	78.66 ± 1.31	209.38 ± 0.71	180.52 ± 10.34	199.24 ± 30.75	171.35 ± 37.52	149.92 ± 31.19	67.96 ± 14.34	146.06 ± 48.04	173.72 ± 39.46	202.50 ± 8.04	366.78 ± 38.31	312.93 ± 26.46	105.31 ± 29.18
Putrescine	-	-	115.67 ± 3.78	59.03 ± 2.70	67.58 ± 3.64	-	108.07 ± 15.41	42.79 ± 8.54	110.54 ± 4.26	99.28 ± 13.57	79.30 ± 10.81	155.95 ± 15.52	256.59 ± 8.92	359.59 ± 80.64	-
Cadaverine	-	-	-	83.38 ± 2.61	100.87 ± 0.44	67.94 ± 1.91	136.90 ± 20.01	-	-	-	-	-	436.03 ± 29.87	252.52 ± 30.31	-
TOTAL	73.87	47.65	194.33	268.41	348.98	267.18	612.10	192.72	178.50	245.34	423.76	358.46	1059.40	925.04	105.31

*: under the detection level (3–5 mg/kg).

**Table 6:** Volatile organic compounds (VOCs) detected by SPME-GC-MS in the samples, expressed as a ratio between the peak area of each molecule and the peak area of the internal standard (4-methyl-2-pentanol). The standard deviation was always below 5%.

VOCs	Italy			Slovenia		Spain							Croatia		
	IM1	IM2	IAL	SN	SWO	ESB	ESE	ESA	ESO	ECB	ECE	ECO	HNS	HS	HZK
Acetone	5.85	3.00	2.61	0.87	1.31	3.70	3.20	- *	1.79	2.26	13.48	4.85	1.86	0.66	1.38
2-butanone	0.55	1.08	1.13	-	0.44	1.42	0.86	-	0.97	47.36	2.70	2.51	94.05	3.50	1.30
2,3-butanedione	1.87	1.54	0.23	0.41	0.48	0.50	2.44	0.18	0.52	5.93	3.68	1.32	2.14	3.42	2.03
2-pentanone	0.73	0.52	0.51	-	-	-	1.45	-	-	-	1.53	-	-	-	-
Methyl Isobutyl Ketone	0.89	0.81	0.70	0.62	0.50	0.64	0.52	0.61	2.87	0.79	2.46	3.00	2.97	3.94	3.90
4-methyl-3-penten-2-one	3.61	3.42	3.51	2.87	2.87	2.98	2.96	2.03	7.43	1.84	6.52	7.72	7.48	5.96	10.08
2,6-dimethyl-4-heptanone	-	-	-	-	-	-	-	-	-	0.65	5.39	6.87	3.20	3.42	-
2-heptanone	1.71	4.15	1.99	1.39	1.02	4.94	2.44	-	3.14	-	4.32	1.70	2.76	-	-
3-octanone	1.10	0.87	0.72	0.41	0.28	0.48	0.34	-	2.88	-	2.65	3.23	1.55	-	1.76
2-octanone	1.13	1.44	0.72	0.86	1.64	0.97	1.08	1.01	1.60	1.32	2.37	2.57	2.03	1.06	0.96
3-hydroxy-2-butanone	3.72	2.85	0.71	10.72	7.98	1.27	9.37	-	1.57	4.19	-	10.74	9.03	-	11.78
2,5-octanedione	0.39	4.64	0.72	1.34	0.78	-	-	-	2.84	-	-	-	14.68	-	-
2-nonanone	1.06	4.40	0.81	-	-	5.74	0.30	-	1.58	-	-	0.70	1.49	-	-
2-undecanone	0.84	0.65	-	0.78	0.71	3.06	0.40	-	-	-	-	-	1.47	2.88	-
**Ketones**	**23.46**	**29.36**	**14.35**	**20.26**	**18.02**	**25.70**	**25.37**	**3.83**	**27.18**	**64.33**	**45.09**	**45.21**	**144.71**	**24.84**	**33.19**
3-methyl-butanal	1.03	0.38	0.73	-	0.29	1.65	1.74	-	-	-	-	-	1.20	-	-
Pentanal	0.44	2.01	0.69	0.70	0.90	0.95	0.74	0.21	4.22	-	-	0.65	6.56	-	-
Hexanal	4.90	38.66	6.59	3.38	3.51	1.56	5.77	1.09	48.94	0.81	3.29	3.44	110.21	-	2.92
Heptanal	-	-	-	-	-	-	-	-	-	-	-	-	6.53	-	-
Octanal	-	-	-	-	-	-	-	-	-	-	2.46	-	5.10	1.47	-
2-heptenal	-	-	-	-	-	-	-	-	-	-	-	-	51.97	-	-
Nonanal	4.01	6.33	4.60	6.75	3.97	4.37	3.45	3.92	6.11	1.03	11.14	8.97	14.11	7.47	7.85
Decanal	1.72	2.59	1.57	-	-	2.63	0.91	1.27	2.01	0.78	-	-	5.16	-	-
Benzaldehyde	3.64	3.99	1.58	2.06	3.18	3.70	1.37	0.56	1.14	3.80	8.98	25.37	38.39	6.26	2.24
Benzeneacetaldehyde	5.47	4.03	14.61	5.30	13.70	6.28	5.66	6.27	4.85	1.30	4.82	2.49	30.31	127.99	23.42
Hexadecanal	1.31	2.93	2.63	1.40	1.61	1.39	1.03	1.10	1.30	3.49	5.58	3.26	-	-	-
**Aldehydes**	**22.52**	**60.92**	**33.00**	**19.59**	**27.17**	**22.54**	**20.67**	**14.42**	**68.57**	**11.21**	**36.27**	**44.18**	**269.54**	**143.19**	**36.43**
Ethyl alcohol	15.46	6.17	20.31	24.81	18.94	101.21	20.17	270.32	173.69	41.81	29.99	18.04	71.23	118.85	52.12
2-butanol	-	-	-	-	-	-	-	-	-	0.49	-	-	4.72	3.87	0.83
1-propanol	-	-	-	-	-	-	-	-	-	9.77	4.51	-	6.49	14.93	-
2-propen-1-ol	-	-	-	0.67	0.46	-	-	-	-	0.33	1.42	1.29	3.22	-	0.41
Isoamyl alcohol	0.66	0.44	0.35	0.49	0.40	2.10	3.45	6.19	2.66	0.51	2.13	1.03	3.72	-	1.73
1-pentanol	0.43	1.04	0.87	0.79	0.41	2.07	0.91	-	1.21	-	-	0.67	5.46	-	-
1-hexanol	2.08	3.88	2.00	1.69	2.26	1.89	5.01	2.68	8.71	6.98	5.48	3.96	-	3.00	-
1-octen-3-ol	2.85	5.01	1.30	0.62	1.08	0.49	1.41	-	2.81	0.44	2.70	1.19	19.92	1.52	0.84
1-octanol	0.69	0.88	0.67	0.81	0.62	0.55	0.94	0.52	0.61	0.41	1.36	0.93	3.09	1.26	1.03
Benzyl alcohol	0.94	-	0.37	0.85	0.93	1.06	0.91	0.82	1.08	3.43	2.12	4.85	43.88	11.04	-
Phenylethyl alcohol	1.16	1.17	0.59	2.26	3.40	1.62	3.01	7.85	1.70	-	4.11	2.84	5.05	28.23	1.45
**Alcohols**	**24.27**	**18.59**	**26.46**	**32.99**	**28.50**	**111.00**	**35.81**	**288.37**	**192.46**	**64.17**	**53.81**	**34.81**	**166.79**	**182.70**	**58.40**
Acetic acid	11.28	25.95	27.28	95.75	79.25	87.10	14.38	11.72	65.64	203.95	349.10	185.04	73.10	114.92	16.40
Propanoic acid	0.43	0.71	0.43	2.93	0.87	1.10	0.48	-	-	7.07	7.65	0.94	14.39	22.71	-
Butanoic acid	10.88	5.70	4.23	10.48	8.37	15.04	9.36	19.13	20.17	4.91	14.94	20.07	4.68	8.52	3.43
Isovaleric acid	8.56	3.06	0.76	1.39	1.43	4.11	2.89	1.03	3.99	2.48	4.35	2.74	-	-	3.38
Pentanoic acid	0.68	0.78	0.59	1.08	0.87	1.07	0.91	0.68	1.17	1.21	2.90	1.11	0.95	2.82	1.00
Hexanoic acid	2.98	3.25	2.62	5.04	4.12	5.30	6.03	2.74	13.22	6.21	16.35	6.68	16.21	5.94	2.60
4-hexenoic acid	-	-	-	-	-	-	-	-	-	54.84	-	-	-	-	-
Heptanoic acid	0.89	1.18	0.74	1.32	1.08	1.41	0.98	0.66	1.45	1.60	2.22	1.32	1.30	1.86	1.06
Octanoic acid	2.55	3.14	2.65	6.45	4.63	3.84	3.89	2.63	6.19	7.34	5.93	4.90	4.57	6.79	2.76
Nonanoic acid	1.99	2.01	1.75	2.64	1.91	2.45	1.22	1.94	2.27	1.81	2.28	3.03	3.58	3.62	2.01
n-decanoic acid	1.82	2.98	2.56	6.68	5.18	5.58	2.52	2.35	4.38	7.37	5.86	5.09	4.84	6.10	1.79
Dodecanoic acid	1.70	4.24	1.99	8.87	1.99	4.81	0.89	2.23	2.36	4.10	1.66	1.87	1.50	2.67	0.84
**Acids**	**43.75**	**52.98**	**45.61**	**142.64**	**109.69**	**131.80**	**43.55**	**45.12**	**120.82**	**302.91**	**413.24**	**232.80**	**125.11**	**175.96**	**35.27**
Acetic acid, methyl ester	-	-	-	-	-	-	-	-	-	2.74	9.55	2.73	-	1.72	-
Ethyl Acetate	1.30	1.02	2.93	2.78	2.42	23.96	1.60	12.85	22.98	14.30	6.52	3.31	2.90	13.65	1.07
Butanoic acid, ethyl ester	-	-	0.33	0.91	0.72	1.94	0.78	4.71	5.68	0.90	0.97	0.46	1.00	8.84	-
Hexanoic acid, ethyl ester	2.58	1.80	1.73	1.79	1.76	4.17	2.53	6.97	17.18	2.72	1.71	-	-	7.58	-
4-Hexenoic acid, ethyl ester	-	-	-	-	-	-	-	-	-	37.68	-	-	-	-	-
Octanoic acid, ethyl ester	-	-	1.14	1.60	1.07	7.61	1.12	14.60	13.55	6.24	4.20	-	2.89	7.18	1.00
Dodecanoic acid, methyl ester	-	-	-	-	-	-	-	-	-	1.45	2.28	0.61	-	-	-
Dodecanoic acid, ethyl ester	1.75	-	1.35	1.84	1.03	7.95	0.62	13.02	6.98	4.65	2.56	-	2.63	9.86	-
Benzoic acid, ethyl ester	-	-	-	-	-	-	-	-	2.82	11.28	-	-	-	-	-
**Esters**	**5.62**	**2.82**	**7.48**	**8.93**	**7.00**	**45.64**	**6.65**	**52.15**	**69.20**	**81.96**	**27.79**	**7.11**	**9.42**	**48.82**	**2.07**

* not detected under the adopted conditions.

## Data Availability

Not applicable.

## Supplementary Materials

The following are available online at https://www.mdpi.com/article/10.3390/foods10112691/s1, Table S1: Volatile organic compounds (VOCs) detected by SPME-GC-MS deriving from spices or allium. Results are expressed as a ratio between the peak area of each molecule and the peak area of the internal standard (4-methyl-2-pentanol). The standard deviation was always below 5%. Table S2: Volatile organic compounds (VOCs) detected by SPME-GC-MS deriving from smoking. Results are expressed as a ratio between the peak area of each molecule and the peak area of the internal standard (4-methyl-2-pentanol). The standard deviation was always below 5%.
