# Supplementary material for: Mediterranean Spontaneously Fermented Sausages: Spotlight on Microbiological and Quality Features to Exploit Their Bacterial Biodiversity
Source: Foods. 2021 Nov 3;10(11):2691. doi: 10.3390/foods10112691 (PMC8624356; doi:10.3390/foods10112691)
Supplement: Supplementary file 1 [file foods-10-02691-s001.zip › foods-1450408-supplementary.pdf]

Table S1: Volatile organic compounds (VOCs) detected by SPME-GC-MS deriving from spices or allium. Results are expressed as ratio between peak area of each molecule and peak area of the internal standard (4-methyl-2-pentanol). The standard deviation was always below 5%.

**VOCs**		**Italy**			**Slovenia**		**Spain**							**Croatia**		
		**IM1**	**IM2**	**IAL**	**SN**	**SWO**	**ESB**	**ESE**	**ESA**	**ESO**	**ECB**	**ECE**	**ECO**	**HNS**	**HS**	**HZK**
Allium compounds	Ethylene sulfide	-	-	-	89.60	46.31	-	-	-	-	6.04	60.08	20.89	-	20.45	48.28
	Allyl methyl sulfide	1.33	-	-	11.88	8.60	6.72	-	-	0.71	4.36	12.68	28.77	-	4.66	46.80
	Dimethyl disulfide	-	-	-	-	-	-	-	-	-	-	-	-	-	36.33	-
	Diallyl sulfide	-	-	-	-	-	-	-	-	-	-	25.88	-	-	4.49	-
	Diallyl disulfide	-	-	-	39.33	33.85	-	-	0.68	0.92	4.86	60.72	22.00	7.58	9.56	48.09
Terpenes	α-Pinene	31.94	21.25	2.42	12.63	9.91	27.47	1.49	8.08	4.5	-	-	1.54	-	-	16.51
	β-Pinene	31.86	15.47	1.33	13.91	13.21	25.03	1.17	9.57	4.99	-	-	1.21	-	-	23.82
	β-Phellandrene	100.36	54.25	3.82	25.24	17.98	45.89	3.05	36.93	14.53	-	-	2.16	-	-	13.42
	α-Phellandrene	14.71	6.34	-	25.63	21.42	26.41	1.14	4.87	5.48	0.71	-	-	2.89	1.31	36.01
	3-carene	26	9.64	-	62.75	59.17	58.11	0	20.26	14.94	-	-	-	-	-	34.2
	β-Myrcene	59.91	32.93	5.86	46.22	36.95	54.16	2.63	26.22	4.4	-	-	-	-	-	37.5
	β-Pinene	3.65	1.65	-	5.32	4.63	4.72	-	2.14	-	-	-	-	-	-	-
	4-carene	20.51	8.43	4.48	16.73	14.31	18.04	5.67	11.44	11.85	-	-	9.36	-	-	21.86
	D-limonene	196.37	110.64	7.22	141.89	123.93	161.15	9.64	87.79	57.08	2.06	2.85	8.5	3.43	3.51	222.73
	β-ocimene	8.53	4.29	0.34	2.66	2.19	4.73	-	1.03	0.73	-	-	-	-	-	2.1
	3-carene	6.47	2.68	5.35	2.14	1.7	5.22	2.96	6.49	2.81	0.27	-	7.52	-	-	2.46
	P-cymene	13.35	6.63	5.46	14.28	12.53	13.75	3.76	14.26	8.97	-	-	-	-	-	20.31
	cis- β-Terpineol	2.26	1.3	0.49	0.39	0.38	1.82	0.18	2.43	1.55	-	-	1.77	-	-	-
	α-Cubebene	2.25	0.95	-	1.15	1.13	1.89	0.67	1.84	1.82	-	-	0	-	-	1.9
	Copaene	21.52	7.96	1.07	8.71	8.76	7.66	2.37	10.94	11.04	-	-	1.94	1.27	-	11.78
	Linalool	7.8	3.13	23.89	4.39	3.07	2.88	0.96	4.41	3.8	-	-	2.59	-	-	4.55
	cis- β-Terpineol	2.2	1.91	0.73	0.88	0.66	1.96	0.53	3.05	2.07	-	-	3.99	-	-	-
	3,5-octadien-2-one	-	-	-	-	-	-	-	-	-	-	-	-	1.45	7.27	-
	trans- α-bergamotene	7.66	5.74	-	-	-	4.75	-	0.64	-	-	-	-	-	-	-
	Caryophyllene	149.65	52.03	6.28	70.26	68.19	65.75	11.9	37.68	54.73	-	-	7.18	2.52	-	84.85
Phenyl propen es	Safrole	-	-	-	-	-	45.90	-	45.82	1.79	-	-	-	-	-	-
	Methyl eugenol	-	-	-	-	-	-	-	8.94	0.69	-	-	-	-	-	-
	Eugenol	-	-	18.11	0.89	0.76	1.26	-	2.80	0.81	1.46	-	1.77	-	-	-
	**Total**	**708.33**	**347.22**	**86.85**	**596.88**	**489.64**	**585.27**	**48.12**	**348.31**	**210.21**	**19.76**	**162.21**	**121.19**	**19.14**	**87.58**	**677.17**

*not detected under the adopted conditions.

Table S2: Volatile organic compounds (VOCs) detected by SPME-GC-MS deriving from smoking. Results are expressed as ratio between peak area of each molecule and peak area of the internal standard (4-methyl-2-pentanol). The standard deviation was always below 5%.

**Smoking VOCs**	**Italy**			**Slovenia**		**Spain**							**Croatia**		
	**IM1**	**IM2**	**IAL**	**SN[§]**	**SWO[§]**	**ESB**	**ESE**	**ESA**	**ESO**	**ECB[†]**	**ECE[†]**	**ECO[†]**	**HNS**	**HS[§]**	**HZK**
2-furanmethanol	-*	-	-	7.26	7.02	-	-	-	-	6.16	2.85	1.52	2.28	51.82	-
Furfural	-	-	-	-	-	-	-	-	-	-	-	-	-	4.71	-
2-methoxy-Phenol	-	-	-	7.06	5.90	-	0.48	0.57	-	13.42	8.04	4.75	1.57	87.49	-
2-methoxy-4-methyl-Phenol	-	-	-	2.84	2.42	-	-	-	-	4.16	2.85	1.99	-	28.91	-
2-methyl-Phenol	-	-	-	3.24	2.66	-	-	-	-	3.09	2.68	1.60	0.91	20.04	-
Phenol	-	-	-	11.63	7.35	-	-	-	-	8.30	5.51	2.89	2.10	52.45	-
4-ethyl-2-methoxy-Phenol	-	-	-	-	-	-	-	-	-	2.08	2.01	1.52	-	10.07	-
2,4-dimethyl-Phenol	-	-	-	1.26	1.27	-	-	-	-	1.93	-	-	-	7.60	-
4-methyl-Phenol	0.64	-	0.54	2.32	1.95	1.50	-	-	1.25	3.42	1.81	1.95	1.01	13.92	-
3-methyl-Phenol	-	-	-	3.30	2.67	1.09	-	-	-	3.68	2.57	1.85	-	18.97	-
2,6-dimethoxy-Phenol	-	-	-	-	-	-	-	-	-	-	-	-	-	9.90	-
**Total**	**0.64**	**-**	**0.54**	**31.64**	**24.23**	**2.59**	**0.48**	**0.57**	**1.25**	**40.07**	**25.48**	**16.55**	**5.59**	**249.35**	**-**

*: not detected under the adopted conditions.

[§]: samples subjected to a smoking phase.

[†]: samples added with smoked paprika.
